# Migrations in Latin America: the corridor to the American dream is a public health problem

**DOI:** 10.15446/rsap.V25n3.114016

**Published:** 2024-05-01

**Authors:** Francisco Camargo-Assis, Yeimi Lopez-Mejia, Ameth Salim-Mattar, Salim-Mattar

**Affiliations:** 1 FC: Anesthesiol. Spec. Critical Medicine and Intensive Care. Intensive Care Unit, Clínica Zayma. Montería, Colombia. pcamassis@gmail.com Clínica Zayma Critical Medicine and Intensive Care Intensive Care Unit Clínica Zayma Montería Colombia; 2 YL: Geogr. Spec. Geographic Information Systems. Instituto de Investigaciones Biológicas de los Trópicos, Universidad de Córdoba. Montería, Colombia. Fernandalopezmejia0117@gmail.com Universidad de Córdoba Instituto de Investigaciones Biológicas de los Trópicos Universidad de Córdoba Montería Colombia; 3 AS: MD. Facultad de Medicina, Universidad del Norte. Barranquilla, Colombia. amethm@uninorte.edu.co Universidad del Norte Facultad de Medicina Universidad del Norte Barranquilla Colombia amethm@uninorte.edu.co; 4 SM: Biolog. M. Sc. Microbial Biotechnology. Ph. D. Clinical Microbiology. Instituto de Investigaciones Biológicas de los Trópicos, Universidad de Córdoba. Montería, Colombia. smattar@correo.unicordoba.edu.co Universidad de Córdoba Instituto de Investigaciones Biológicas de los Trópicos Universidad de Córdoba Montería Colombia smattar@correo.unicordoba.edu.co

**Keywords:** Social alienation, transients and migrants, poverty, violence, communicable diseases, travel-related illness *(source: MeSH, NLM)*, Alienación social, transeúntes y migrantes, pobreza, violencia, enfermedades transmisibles, enfermedades relacionadas con los viajes *(fuente: DeCS, BIREME)*

## Abstract

**Introduction:**

Migrant movements have tripled, and it is evident throughout the Americas. On the way to North America, people come from South America, the Caribbean, mainly Haiti, Cuba, Asia, and Africa. People migrate for work, study, humanitarian situations, poverty, violence in their territories, lifestyle change, or family reunification.

**Objetive:**

The aim of this study is to assess the impact of most frequent infectious conditions of the population exposed to migratory movement through the Americas.

**Materials and Methods:**

The present review focuses on the leading infectious diseases migrants acquired during the arduous journey through the tropical areas. Official migration web sides, journals, and scientific journals were used to get information.

**Results:**

Some infectious diseases have re-emerged in transit countries used by migrants, and the increase in migratory phenomena, triggered cases of HIV/AIDS, Tuberculosis, arbovirosis, Malaria, and hepatitis, among others.

**Conclusions:**

Migrants suffer infectious diseases as they pass the countries to the USA; besides, they suffer violence and even sexual assault. The receiving countries should establish public policies to regularize the access of migrants to health services, which include preventive programs and easily accessible vaccination plans. Some of the pathologies suffered by migrants are preventable, although there are groups of populations in extreme social conditions and with compromised nutritional status. The need to establish a primary medical care center to apply an innovative border and transnational protocols for infectious diseases for migrant populations is highlighted.

## RESUMEN

**Introducción:**

Los movimientos migratorios se han triplicado y son evidentes en todo el continente americano. En la ruta hacia América del Norte, las personas proceden de América del Sur, el Caribe, principalmente Haití, Cuba, Asia y África. Las personas emigran por trabajo, estudios, situaciones humanitarias, pobreza, violencia en sus territorios, cambio de estilo de vida o reunificación familiar.

**Objetivo:**

El propósito de este estudio es hacer una valoración del impacto de los cuadros infecciosos más frecuentes sobre la población expuesta a los movimientos migratorios a través de las Américas.

**Materiales y Métodos:**

La presente revisión se centra en las principales enfermedades infecciosas que adquieren los migrantes durante el arduo viaje a través de las zonas tropicales. Para obtener información se utilizaron páginas web oficiales sobre migración, diarios y revistas científicas.

**Resultados:**

Algunas enfermedades infecciosas han resurgido en los países de tránsito empleados por los migrantes, y el aumento del fenómeno migratorio disparó los casos de VIH/SIDA, tuberculosis, arbovirosis, malaria y hepatitis, entre otras.

**Conclusiones:**

Los migrantes sufren enfermedades infecciosas en su paso por los países hacia los Estados Unidos, además de sufrir violencia e incluso agresiones sexuales. Los países receptores deben establecer políticas públicas para regularizar el acceso de los migrantes a los servicios de salud, que incluyan programas preventivos y planes de vacunación de fácil acceso. Algunas patologías que sufren los migrantes son prevenibles, aunque existen grupos de población en condiciones sociales extremas y con un estado nutricional comprometido. Se destaca la necesidad de establecer un centro de atención médica que aplique protocolos fronterizos y transnacionales innovadores para las enfermedades infecciosas de las poblaciones migrantes.

Since 1970, migrants in the world have grown exponentially. Migrant movements have tripled, and it is evident throughout the Americas. On the way to North America, people come from South America, the Caribbean, mainly Haiti, Cuba, Asia, and Africa. International migrants are defined as those who move from their place of residence across the geographical limits of countries, temporarily or permanently 1. People migrate for work, study, humanitarian situations, poverty, violence in their territories, lifestyle change, or family reunification 2. The global migrant population is approximately 280 million people. In Colombia, the migration process is done through traditional transportation but is not strictly supervised by the national transportation authorities. The extreme necessity uses these means of transport to reach the intermediate destination site. Vessels are used without adequate security elements, and there are reports of human tragedies, such as boat wrecks in the Caribbean Sea or buses with fatal mechanical failures 3. Therefore, institutions protecting human rights have requested increased surveillance and controls of all transport vehicles' state of mechanics and permits 3.

## MATERIALS AND METHODS

Method A systemic search was conducted on the infectious diseases acquired by migrants during their journey across the continent. The search consisted of analyzing relevant studies published between 2000 and 2023. The literature search was based on Google Scholar and PubMed, official migration office websites, and national and international press information

## RESULTS

**Figure 1:**
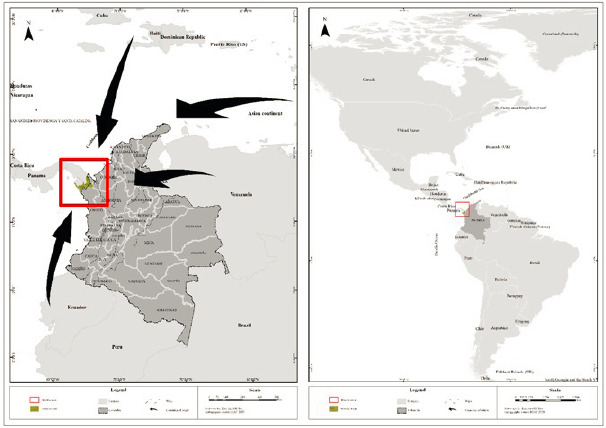
Geographic situation of Colombia and access to different migrant populations

The human mobilizations in Latin America aim to reach the United States of America. To achieve this, they must cross the Darién National Park, on the border between Colombia and Panama, an area dense in forests, inhospitable, and devoid of food and security conditions (Figure 1). Additionally, the roads are shared with drug traffickers and with high rates of sexual and intrafamily violence during the journeys 4.

**Figure 2:**
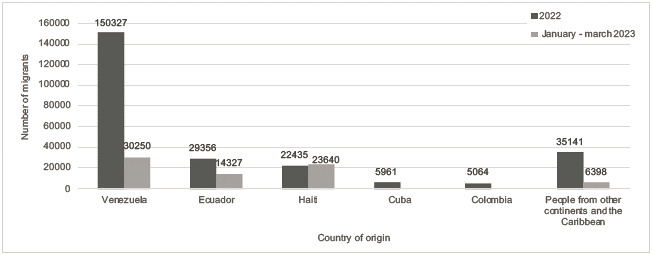
Migrant citizens passing through Colombia to the US. In blue accumulated migrants, 2022. In gray migrants between January-March 2023

In January 2022 in Colombia, the migration of 4 702 people was reported; in the first month of 2023, it amounted to 21,307 migrants, most of them Haitians, Ecuadorians, Venezuelans, Chinese, and Indians. In 2022, 248,284 people crossed the border between Colombia and Panama, 150,327 Venezuelans, 29,356 Ecuadorians; 22,435 Haitians; 5,961 Cubans and 5,064 Colombians, and other people from other continents and the Caribbean (Figure 2).

Recently, a UNHCR report dated April 14, 2023, reported data from the Panamanian authorities, who reported that a record number of more than 100,000 people had been reached crossing the Darién 5-six times more than those who arrived in the same period of 2022. The authorities estimate that if the trend continues, the year 2023 could reach 450,000 migrants. Panama is also facing one of the most challenging crises of the last decade due to an unprecedented shift across the Americas. After 2022 was a record year, in which nearly 250,000 refugees and migrants risked their lives crossing the Darién in search of protection and better opportunities, the three quarter of 2023 points to an increase in the transit of people through this route. (Figure 2). According to the statistics of the National Migration Service of Panama, until September 2023, the main nationalities that crossed the Darién jungle were citizens of Venezuela (30,250), Haiti (23,640), Ecuador (14,327), as well as people from China (3,855), India (2,543), and the children of Haitians born in Chile (2,499) and Brazil (2,072). Other nationalities include people from Colombia, Afghanistan, Cameroon, Somalia, and Peru 5.

Colombian government entities issued alert messages so that the authorities implement actions to attend to the migrant populations in the areas where transit communities must establish medical attention points, assistance for children and senior citizens, as well as controls of the authorities so that migrants do not become victims of migrant smuggling and human trafficking networks. The most vulnerable migrant populations are children, adolescents, pregnant women, nursing mothers, and older adults 2.

**Figure 3:**
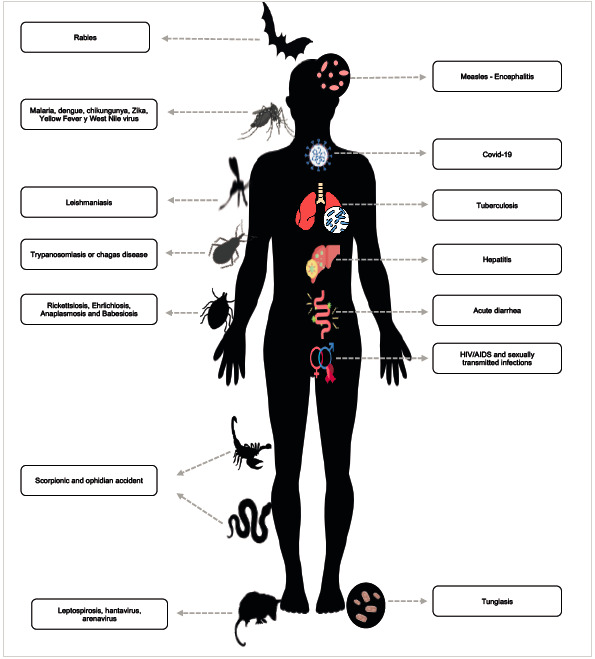
Dynamics of communicable diseases in migrant populations

On the other hand, the dynamics of communicable diseases in migrant populations and their impact on public health are fundamental. The so-called imported diseases are secondary to the migratory phenomenon and refer, in the first place, to those composed of pathologies imported from the country of origin to another destination. Second, those derived from increased transitory population density at border sites. Finally, those acquired during the journey due to exposure to wild animals, vector diseases, stings and bites, complications from traumatic injuries due to violence or on the roads traveled, and sexually transmitted diseases (Figure 3).

Once the population settles in the destination country, the health problems are similar to the autochthonous population. However, more than half of the cases of infectious diseases are not generated in the destination countries due to the lack of vector or intermediate hosts. The surveillance and control conditions are required when they occur, considering that living conditions have changed due to overcrowding and the scarcity of monetary resources.

The public health risk that the migrant population in the host country implies, with some frequency, generates alarms due to the apparent risk of disease transmission to the local inhabitants, which is transformed into discrimination, violation of privacy, and in some cases, violence 4. It is also important to mention that health professionals have adapted and learned about the demographic changes of migration and, in many cases, have become familiar with tropical infections. The disease burden is high, so health care for these populations generates concern and political and social controversy. In many cases, a progression of pathologies requires follow-up to improve quality of life and prognosis. Nevertheless, there are multiple barriers to accessing health services, in some cases due to linguistic difficulties, which hinder the results and limit the application of preventive models for the population 6.

It is essential to highlight that this work shows some of the most prevalent infectious diseases that affect migrants. However, other diseases could also be of public health concern. The most frequent infectious pathologies in migrant populations are described below.

### Tuberculosis

Transmissible infectious disease screening efforts are focused primarily on tuberculosis. The strategies focus on the search for cases established by the health services for migrants and refugees. According to human rights expert organizations, some of these programs by the receiving country have limitations due to stigmatization towards migrants 7. Migrants from countries with a high incidence of tuberculosis, it is estimated that in 2020 9.9 million people became ill with tuberculosis in the world, of which 1.1 million were children, most of the cases in Southeast Asia (43%), Africa (25%), Western Pacific (18%), Eastern Mediterranean (8.3%), America (3.0%) and Europe (2.3%). A quarter of the world population is a carrier of tuberculosis bacillus, but they have not become ill. In 2021, 14,060 cases of tuberculosis were reported in Colombia, 72 cases of foreigners, and 318 cases were relapses 8.

Tuberculosis continues to be a severe public health problem, and vigilance is justified to end this world epidemic 9. Many basic screening programs for active tuberculosis are designed to detect infection and transmissible cases and focus on detecting the disease at the pulmonary level. Surveillance is performed with immuno-diagnostic testing, risk-based assessment, and later confirmatory molecular diagnostic and microbiological studies such as smear microscopy and culture 10.

Many countries have different screening strategies for migrants since they generally travel by land and sea, which takes a long time to reach their destination, under conditions in agglomeration, so there is a greater risk of contagion; therefore, screening would be justified.

### COVID-19

Although vaccination and natural infection have allowed fewer cases in Latin America, the virus continues circulating and mutating. When migrant populations left their countries, they did not have the full doses, much less the vaccination booster. The possibility that SARS-CoV-2 continues to mutate, as with the omicron variant and hundreds of subvariants descended from it, suggests that the vaccines applied up to now must be redesigned against the new variants. It is a scenario that cannot be ruled out, and that would further aggravate the current social situation at the borders 11.

### Measles

The disease continues to be a significant cause of morbidity and mortality, especially in the pediatric population, escalating to a global public health problem due to low vaccination coverage and its reappearance in populations where there was control and eradication due to high levels of immunity, presenting as small outbreaks and sporadic cases until the current level of spread because it is present in more than 160 countries, with infected travelers being the main transmission vehicle 12. Therefore, the application of highly effective vaccination, with almost no contraindications and side reactions 13.

In the recent epidemiological alert of February 2023, the Americas region was declared measles-free in 2016. However, after that period, imported cases from other regions and between countries in the Americas increased 14. The increase in cases was related to the measles outbreaks in Brazil and Venezuela, contributing to 93% of the events reported in Colombia. The risk of occurrence is at its highest point in the last 30 years since vaccination coverage rates continue to decline worldwide, according to data from the World Health Organization (WHO) and the Fund for United Nations Children's Fund (Unicef), more than 2.7 million children under one year of age do not have the full vaccination schedule, corresponding to 20% of children susceptible to vaccine-preventable diseases.

### HIV/AIDS and sexually transmitted infections

Sexually transmitted diseases are a group of diseases that the massive flow of migrants has negatively impacted. In the migrant 15 reported that 1% of the cases of de novo diagnosis of HIV are imported from approximately 12 countries. Humanitarian conflicts facilitate the increase in the incidence of these diseases, mainly due to the absence of health services and gender violence. It must be considered that only 59% of the migrant population with HIV/AIDS have access to antiretroviral therapy, and only 7% have low viral loads 15,16. During the past decade in Colombia, the number of people living with HIV/AIDS has been stable. However, in Venezuela, 70% of the carriers of this disease have not received medication to control the disease, and mortality has increased doubled from 2010 to 2020 15-17.

Additionally, there is a lack of diagnostic tests available, with the consequent underreporting of cases, lack of notification to the health authorities, and the illegal marketing of antiretrovirals in patients with compromised nutritional conditions. Coinfections in HIV/AIDS patients tend to increase; in the case of tuberculosis, patients with acquired immunodeficiency have a risk more significant than 16 times to contract this disease through the respiratory tract, being a lethal combination. In 2021, 187,000 people died of Hiv-associated tuberculosis, and the percentage of notification of patients with tuberculosis that HIV was documented was 76% 17,18. Syphilis, chlamydia, and gonorrhea are also significant sexually transmitted infections among migrants who have unprotected sex.

### Arbovirosis

In the Americas, there is a significant increase in cases of dengue, Chikungunya, Zika, yellow fever, and West Nile virus 19,20. Regarding dengue, the increase in the last four decades has been from 1.5 million cases in the 1980s to 16.2 million in the 2010-2019 decade 19,20. Currently, about 500 million people in this area are at risk of contracting dengue of different circulating serotypes (DENV-1, DENV-2, DENV-3, and DENV-4) 20. In a cohort, most of the viral hemorrhagic fevers correspond to severe dengue infection, and other imported cases are yellow fever pictures, which establish sanitary policies for vector management and limit the transmission of the infection, stimulate, and make compulsory the vaccination schemes in people from tropical areas 19,20. In retrospective series, it has been documented that the clinical characteristics of the migrating populations are different in presenting symptoms and severity of the disease. In the subgroup analysis, some serotypes have a higher proportion of plasmatic leakage, hemorrhagic manifestations, and organic dysfunctions derived from the immunological status, comorbidities, and the predominant serotypes in each area 20-22. Dengue is undoubtedly a disease that occurs in migrants traveling through the Darien, but the prevalence is not known.

Yellow fever *(Flaviviridae)* is a vector-borne disease in tropical regions, and the disease is endemic in 27 countries in Africa, and 13 in the Americas, predominantly in Brazil, Peru, Ecuador, and Colombia. Many cases are asymptomatic, but 15% develop severe disease, with 20-60% mortality. There are no reports of this pathology during migration movements, and case descriptions are presented in temporary international visitors to endemic countries.

Zika virus (ZIKV) is a flavivirus transmitted by the bite of *Aedes aegypti* mosquitoes, by sexual contact, and during pregnancy from mother to fetus. The disease is globally distributed in 87 countries, whose disease outbreaks have occurred since August 2015, with maximum peaks in 2016, and subsequently, the incidence of cases has decreased 23. The virus is implicated in cases of microcephaly in newborns of mothers with Zika virus infection 24. Migratory movements for several decades have allowed the expansion of the Zika virus in jungle areas since the cycle in non-human primates and taken to urban areas in epidemic cycles of human communities suffering from mosquito bites. Other little-studied arboviruses that are present in the tropical migratory corridor are Mayaro (alphavirus), Encephalities equine virus, (Madarriaga), and Oropuche *(Orthobunyaviridae),* among others.

### Malaria

Transborder malaria is defined as the transmission of the disease with the presence of cases that cross borders. The migrant population is left out of control strategies and, resulting in undetected or untreated infections. Cases of malaria in patients from other countries, mainly due to Plasmodium falciparum, appear in the first three months after arrival; this means there is a significant population of asymptomatic patients at the time of entry to the host country

The information on pediatric patients is scarce, approximately 10% of the migrant population are pediatric patients, and in studies that describe the cases of children who arrive in the process of migration with a febrile syndrome, it was determined that 56% were patients without a specific focus, 15.4% with respiratory disease, 11.7% with acute diarrhea, and of all cases, 7.4% required management in the intensive care unit. No specific etiology was determined in 24% of the cases, but the diagnosis was malaria in 29.4% of the patients. There is an increase of more than 90% in malaria cases in Colombia due to the massive migrant population from Venezuela that has arrived in Colombia 25.

### Tick-borne diseases

Ticks and mosquitoes are the most common vectors in the tropics. Among the significant tick-borne diseases is Rickettsia, which are Gram-negative, obligate intra-cellular bacteria that infect various types of cells of the arthropod host and human endothelial cells. Are classified into several groups: typhus group, which includes louse-borne *R. prowazekii,* which causes epidemic typhus, and flea-borne Rickettsia typhi, which causes endemic typhus. In addition, due to the overcrowding suffered by migrants in shelters and makeshift tents, transmission by lice can trigger outbreaks of typhus, whose etiological agent is *Rickettsia prowaseki*^26^.

### Leishmaniasis

It is another important parasitosis to which migrants are exposed, and abundant insects transmit it in the tropical rain forests of Colombia and Panama. The parasitic protozoan is transmitted by the bite of infected female sandflies *Lutzomyia* and lodges in the tissues of a vertebrate host 27. The parasite also affects domestic animals such as dogs, cats, and rodents. The cutaneous-mucosal form is the most common, the incidence of leishmaniasis is unknown because there is no monitoring of the diseases acquired during the journey of migrants through jungles and tropical forests 27.

### Acute diarrheal disease

Since migrants must travel many kilometers, their food depends on the supply through migratory routes. Those expose them to foodborne illnesses due to ingesting food prepared and handled in poor sanitary conditions. Noninvasive osmotic diarrhea can be self-limited or treated with fluid and electrolyte replacement, and invasive diarrhea requires antimicrobial treatment. The microorganisms that most affect the migrant population include bacteria, viruses, and parasites. Among bacteria, different *E. coli* serotypes (enterotoxigenic, enteropathogenic, enterohemorrhagic), *Salmonella, Shigella,* and *Campylobacter* affect migrants. Rotavirus, Norovirus, and Adenovirus are involved, and parasites as *Entamoeba, Giardia, Cryptosporidium,* and *Ascaris.* The prevalence, morbidity, and mortality caused by these microorganisms in migrants are unknown 28.

### Leptospirosis

Due to the precarious hygienic conditions of the shelters where the migrants temporarily live, the presence of rodents is frequent. Synanthropic rodents such as *Rattus rattus* and *Mus musculus* can be reservoirs of leptospira. Leptospirosis affects organs such as the kidney, lungs, and cardiovascular system. In addition to leptospirosis, rodent-borne diseases such as Hantavirus and Arenavirus can affect migrants.

With the increase in migratory phenomena, some infectious diseases have re-emerged, varying prevalences according to the place of origin. Because the countries needed to prepare to receive thousands of people, the inadequacy of medical attention soon became apparent. Some of the pathologies suffered by migrants are preventable, although there are groups of populations in extreme social conditions and with compromised nutritional status. The need to establish a primary medical care center to apply an innovative border and trans-national protocols for infectious diseases for migrant populations is highlighted. This makes it possible to screen for infectious pathologies, which reduces complications and sequelae, and simultaneously to contain the disease early. The pharmacological treatment of migrants is challenging because they must move quickly across borders. Therefore, they need to complete the therapeutic scheme, or it is impossible to apply them.

On the other hand, they are issuing suggestions to the receiving countries so that they establish public policies to regularize the access of migrants to health services, which include preventive programs and easily accessible vaccination plans. In this way, contagion can be stopped by potentially preventable infections. Finally, genetic mutations of microorganisms or introduction of new etiological agents across borders should not be ruled out. Therefore, epidemiological surveillance must be carried out, including molecular studies of pathogens and seroprevalence, to monitor the dynamics of border infections ♣
